# Improved Range Estimation Model for Three-Dimensional (3D) Range Gated Reconstruction

**DOI:** 10.3390/s17092031

**Published:** 2017-09-05

**Authors:** Sing Yee Chua, Ningqun Guo, Ching Seong Tan, Xin Wang

**Affiliations:** 1 School of Engineering, Monash University Malaysia, Jalan Lagoon Selatan, 47500 Bandar Sunway, Selangor, Malaysia; csingyee@gmail.com (S.Y.C.); anthony.guo@monash.edu (N.G.); 2 Faculty of Engineering, Multimedia University, Jalan Multimedia, 63000 Cyberjaya, Selangor, Malaysia; cstan@mmu.edu.my

**Keywords:** TOF range imaging, range gated imaging, 3D reconstruction

## Abstract

Accuracy is an important measure of system performance and remains a challenge in 3D range gated reconstruction despite the advancement in laser and sensor technology. The weighted average model that is commonly used for range estimation is heavily influenced by the intensity variation due to various factors. Accuracy improvement in term of range estimation is therefore important to fully optimise the system performance. In this paper, a 3D range gated reconstruction model is derived based on the operating principles of range gated imaging and time slicing reconstruction, fundamental of radiant energy, Laser Detection And Ranging (LADAR), and Bidirectional Reflection Distribution Function (BRDF). Accordingly, a new range estimation model is proposed to alleviate the effects induced by distance, target reflection, and range distortion. From the experimental results, the proposed model outperforms the conventional weighted average model to improve the range estimation for better 3D reconstruction. The outcome demonstrated is of interest to various laser ranging applications and can be a reference for future works.

## 1. Introduction

Because of the non-contact and non-destructive nature, laser has been a favoured solution especially in remote sensing and machine vision [1]. While a variety of techniques are suited for different applications, the range gated technique provides efficient laser ranging based on the Time-of-Flight (TOF) principle where the distance is determined from the travel time measured between the emitted and reflected laser pulse [2]. In recent years, this technique has become even more cost effective with the continuous development of equipment and the processing method. Due to good application prospects, the range gated technique has been widely applied for target detection and recovery [3], night vision [4], underwater [5], and 3D imaging [6].

In spite of the advancement in equipment, i.e., laser and sensor technology, the accuracy of 3D range gated reconstruction remains a challenge due to various factors in the system. Therefore, range estimation improvement is crucial to accomplish accurate reconstruction. A laser signal reflected from the target surface can be exploited using different range estimation algorithms such as peak estimator/discriminator, leading edge detection, and average (centre of mass/gravity determination) [7]. In some applications, the use of matched filter (cross-correlation) and maximum likelihood estimator are possible to provide better range estimation based on a reference signal or model [8,9]. Peak detection determines the range by the maximum of the returned signal where the highest power theoretically coincide with the reflected target. Leading edge detection works based on a predefined threshold where the range is determined by the threshold crossing in the returned signal [10]. Matched filter compares the received signal to a reference using a cross-correlation technique. Maximum likelihood estimator performs similarly to a matched filter with respect to the mean squared error (MSE) but has a bias in range estimation when a signal deviates from the model assumed [9].

The range gated technique utilises the reflected time slices that consist of the reflectivity and range information simultaneously for 3D reconstruction. There are some practical complications to be considered. Although the reflected laser pulse is a Gaussian function in general, the actual signal returned is not precisely known since the target scene or object of interest is unknown. In this context, peak estimator and leading edge detection are clearly not suitable due to the noisy nature of the reflected laser signal recorded in the image pixels [11]. As for matched filter and maximum likelihood method, a reference signal or model needs to be assumed that is not feasible in some cases. Moreover, inaccurate reference directly affects the range accuracy. Because of the aforementioned considerations and limitations, weighted average method is commonly applied for range estimation in 3D range gated reconstruction [12,13]. However, the range accuracy of this model is influenced by the intensity variation [14].

Essentially, the received intensity is affected by a few main components: laser source, sensor, target, and atmospheric effect [15,16]. In addition, the intensity data is also influenced by other factors such as the near-distance effect [17], multiple returns [18], system settings [19], and automatic gain control [20]. Various parameters and processing techniques were reviewed in literatures [14]. A laser based solution is complicated and requires considerable post-processing efforts because of the characteristics of laser and its changes along the transmission. The laser beam propagation towards the target and return back to the receiver can be described by the spatial-temporal distribution. Since the signal to noise ratio (SNR) is proportional to the laser intensity in general, the ranging performance exhibits a correlated variation to the laser optical power distribution, which is non-uniform profile in practice [21]. System performance is often limited by the reduced SNR due to the decreasing reflected intensity over distance, which restricts the operating range of many applications [22]. Target reflection affects the detection and ranging behaviours that are crucial especially to applications such as target recognition and object modelling [23,24]. These characteristics of target reflection can be significant important to analyse and solve the complex ranging problem [25]. Laser illumination and optical components introduce additional effects which could affect the image formation [26]. As the accuracy of depth and 3D reconstruction strongly relies on the range information, it is necessary to understand the range distortion due to the inhomogeneity of illumination and propose an appropriate correction. Recovery of the actual target echo pulse is a well known problem in range gated systems. The influence of the laser profile [27], distance interference [28], sensor [29,30], and scattering effects [31] has been discussed in various literatures.

This paper provides an in-depth analysis of the 3D range gated reconstruction and proposes a new range estimation model to alleviate the effects induced by the influence factors. In Section 2, system set-up, operating principle, and technique used in 3D range gated reconstruction are described. In Section 3, we discuss the range accuracy in a range gated system with time slicing reconstruction and the associated influence factors. In order to improve the range accuracy, we derive and propose a new range estimation model in Section 4 by considering multiple influence factors in the system. In Section 5, the proposed range estimation model is validated and discussed. Lastly, conclusion is given in Section 6.

## 2. 3D Range Gated Reconstruction

**Figure 1:**
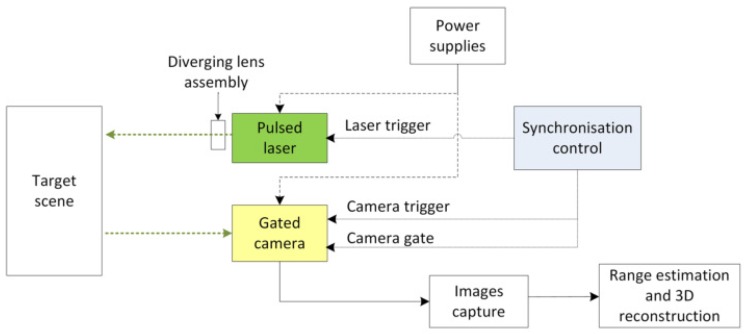
Schematic diagram of range gated imaging system setup.

Figure 1 shows the schematic diagram of a range gated imaging system for 3D reconstruction. The system set-up includes a pulsed laser module, Intensified Charged Coupled Device (ICCD) gated camera and its control unit, delay generator for system triggering and synchronisation, lens assemblies, and power supplies. Additionally, interfaces such as a frame grabber or digital video converter and measurement devices such as a photodetector and oscilloscope are used. The laser and camera are controlled simultaneously by the delay generator during the acquisition of range gated images. A Q-switched Nd:YAG pulsed laser with wavelength 532 nm provides illumination to the target and an ICCD camera is configured to capture the reflected intensity images that are the input for range and 3D reconstruction.

**Figure 2:**
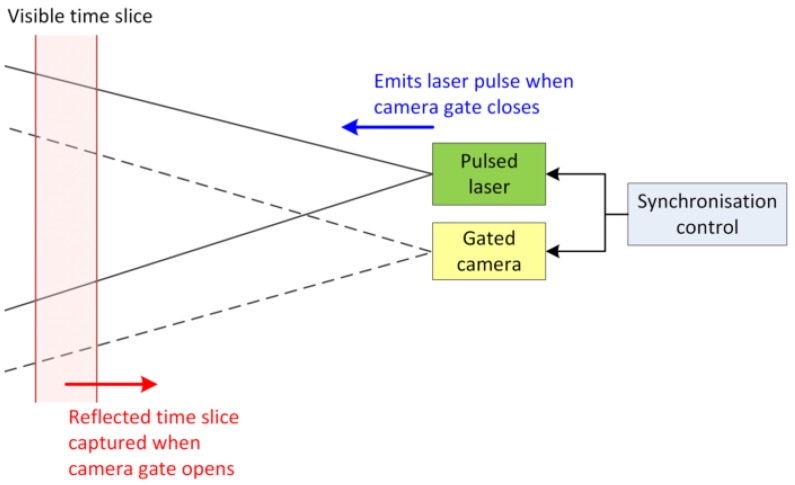
Synchronisation of the laser and camera during range gated image acquisition.

Delay generator is configured to trigger the camera gate to open for a very short duration normally in nanoseconds or picoseconds at the designated delayed time to capture the reflected laser pulse in the form of a two-dimensional (2D) intensity image. Synchronisation between the laser and the gated camera is particularly important during the image acquisition. The camera gate remains closed when the laser pulse is emitted towards the target. The camera gate is configured to open at the designated delayed time to capture the visible time slice reflected in the form of an intensity image. This operating principle of the image acquisition is illustrated in Figure 2.

**Figure 3:**
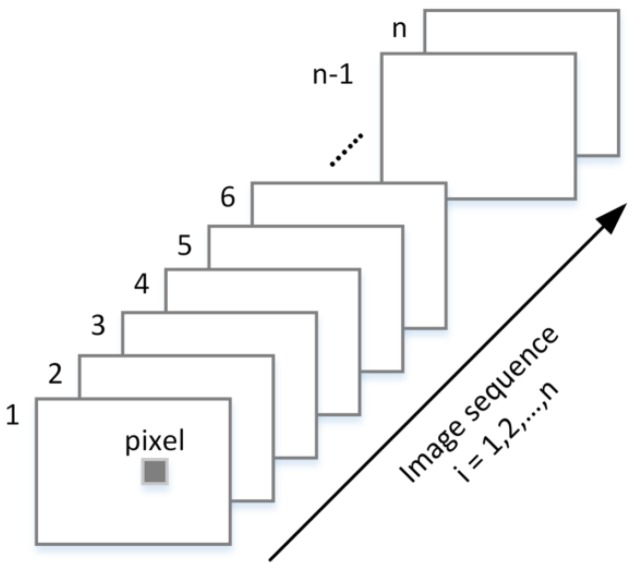
Time slicing technique captures a sequence of intensity images for 3D reconstruction.

Based on the time slicing technique, a sequence of 2D images i=1,2,…,n is acquired by sliding the camera gate $t_{gate}$ at delay time $t_{i}$ with a time step $t_{step}$ to sample range slices from the target scene as illustrated in Figure 3. These images acquired consist of reflectivity and range information simultaneously, which are important for 3D reconstruction.

For each image pixel (x,y), the corresponding range <r>(x,y) can be obtained from the average TOF or two-way travel time <t>(x,y) to construct a 3D depth map for the entire image field-of-view (FOV).

(1) $$<r>\left(x,y\right)=\frac{c<t>(x,y)}{2}.$$

The average TOF <t>(x,y) can be determined from the pixel intensity $I_{i}\left(x,y\right)$ captured over the image sequence acquired using the weighted average method.

(2) $$<t>\left(x,y\right)=\frac{\sum_{i=1}^{n}I_{i}\left(x,y\right)t_{i}}{\sum_{i=1}^{n}I_{i}\left(x,y\right)}.$$

Eventually, the calculated 3D depth map and 2D image textures are used for 3D reconstruction of the target scene.

## 3. Accuracy Analysis

### 3.1. Range Accuracy

In a range gated system with time slicing reconstruction, SNR is expressed in terms of the reflected laser intensity $I_{i}$ and associated noises $\delta I_{i}$ from a sequence of image slices $I_{i}\left(x,y\right)$.

(3) $$SNR=\frac{\sum_{i}I_{i}}{\sqrt{\sum_{i}{\left(\delta I_{i}\right)}^{2}}}.$$

By considering random noise, where ${\left(\delta I_{i}\right)}^{2}\approx I_{i}$, SNR can be simplified as:

(4) $$SNR\approx \frac{\sum_{i}I_{i}}{\sqrt{\sum_{i}I_{i}}}=\sqrt{\sum_{i}I_{i}}.$$

Theoretically, SNR can be estimated from the system parameters as follows:

(5) $$SNR\approx \sqrt{\sum_{i}I_{i}}=\sqrt{\frac{\sigma }{t_{step}}max\left(I_{i}\right)},$$

where the total intensity in an image pixel $I=\sum_{i}I_{i}$ is contributed by number of time slices, which is given by the $\sigma /t_{step}$ factor. Variance of the measured travel time $\sigma^{2}$ depends on the laser pulse width and camera gate time, while $t_{step}$ is the delay time step used for images acquisition. Accordingly, range accuracy is estimated as [12]:

(6) $$\delta r\approx \frac{1}{2}\frac{c\sigma }{SNR}.$$

As can be seen, range accuracy is governed by two parameters: σ and SNR. In general, σ is affected by the system specification, i.e., laser and camera, where the range accuracy can be improved by the hardware advancement. On the other hand, SNR is proportional to the reflected laser intensity, which can be influenced by other conditions such as distance and target reflection.

### 3.2. Influence Factors Affecting Range Accuracy

The reflected laser intensity captured in an image pixel $I_{i}$ is the incident energy of the laser pulse $P_{r}$ integrated when the camera gate G(t) opens, which is expressed as:

(7) $$I_{i}=\int P_{r}\left(t-\frac{2r}{c}\right)G\left(t-t_{i}\right)dt.$$

The reflected laser $P_{r}\left(t\right)$ is delayed by the round trip travel time $\frac{2r}{c}$ and the camera gate G(t) is delayed by time $t_{i}$. The reflected laser energy and its dependencies on multiple influence factors can be defined by the Laser Detection And Ranging (LADAR) equation [32]:

(8) $$P_{r}=\frac{\eta_{sys}\eta_{atm}D^{2}\rho AP_{t}}{r^{2}\theta_{R}{\left(\theta_{t}r\right)}^{2}},$$

where $P_{t}$ represent the transmitted laser energy across range *r*. $\eta_{sys}$ and $\eta_{atm}$ are the system efficiency and atmospheric transmission loss factor, respectively. *D* is the receiver aperture’s diameter and ρ is the target surface reflectivity. $\theta_{t}$ represents the laser transmitter beam diameter and angular divergence. $\theta_{R}$ is the solid angle over which radiation is dispersed upon reflection.

To simplify the LADAR equation, we assume a well-resolved target where the target area *A* equals the projected area of the laser beam. This can be written as:

(9) $$A=\frac{\pi \theta_{t}^{2}r^{2}}{4}.$$

Accordingly, Equation (8) becomes:

(10) $$P_{r}=\frac{\pi \eta_{sys}\eta_{atm}D^{2}}{4r^{2}}\frac{\rho }{\theta_{R}}P_{t}.$$

System efficiency $\eta_{sys}$, atmospheric transmission loss factor $\eta_{atm}$, and the receiver parameter *D* can be regarded as constants under the same set-up condition. On the other hand, distance *r* changes along the laser propagation and acquisition for different time slices. The reflected laser energy $P_{r}$ underlies an inverse range-squared dependency that decreases the intensity captured in the range gated imaging system. The relationship between reflected laser intensity and distance factor was studied in previous work [28]. As a result, SNR decreases with distance and causes a higher range error as deduced from Equation (6). As for $\frac{\rho }{\theta_{R}}$, these parameters vary across image pixels, attributed to the target reflection characteristics. The effect of target reflection to ranging performance was investigated in previous work [33]. The reflected laser intensity is proportional to the target surface reflectivity and the amplitude is maximum when angle of incidence θ=0 and decreases accordingly with the increase in angle. Range accuracy has dependency on the SNR, which is proportional to the reflected laser intensity.

**Figure 4:**
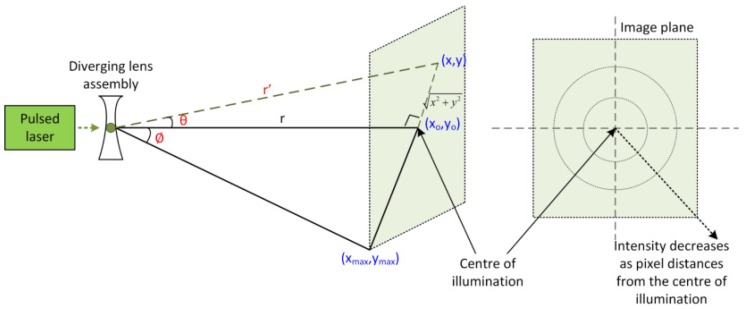
Illustration of range distortion.

Laser illumination with a diverging lens assembly introduces additional effects on the illuminated scene, reflection from the target, and image formation. As illustrated in Figure 4, the laser beam is diverged to cover a target area within $\left(x_{max},y_{max}\right)$ with half diverging cone propagation of angle ϕ. *x* and *y* represent the horizontal and vertical position of an image pixel with diverging angle θ.

Deficiency in straight lines transmission has direct effect on the reflection and image geometry. The difference between orthogonal distance *r* and radial distance $r^{\prime }$ leads to the range distortion. Radial distance $r^{\prime }$ can be written as:

(11) $$r^{\prime }=\sqrt{x^{2}+y^{2}+r^{2}}.$$

Orthogonal distance *r* is normally used, assuming that the illumination is at the centre or perpendicular to the image plane. This is the ideal condition where the laser approximates to directional lighting. In reality, pixels within the angular space close to the illumination centre most likely receive maximum reflection while other pixels exhibit variation due to the illuminant direction [26,34]. This results in inhomogeneous illumination where intensity decreases as the pixels distance from the centre of illumination and causes the range distortion.

## 4. Proposed Range Estimation Model

Theoretically, the system performance can be optimised if the effect of influence factors can be fully compensated in the range algorithm. Therefore, we propose a new range estimation model in this section. Specular and diffuse reflection are the fundamental reflection mechanisms that are practically involved in any target surface. We apply the Bidirectional Reflection Distribution Function (BRDF) model to describe the reflection behaviours due to the interference of multiple parameters [35] as follows:

(12) $$BRDF=BRDF_{S}+BRDF_{D}=\frac{K_{S}}{cos^{6}\theta }exp\left(\frac{-tan^{2}\theta }{s^{2}}\right)+K_{D}cos^{m}\theta ,$$

where $BRDF_{S}$ and $BRDF_{D}$ denote the specular and diffuse component, respectively. $K_{S}$ and $K_{D}$ are the specular and diffuse reflection constants, θ is the angle of incidence and reflection, *s* is the surface slope, and *m* is the diffusivity coefficient. These parameters can be estimated from the reflected laser intensity with respect to the angle of incidence. The BRDF parameters vary depending on the target surface properties and reflection characteristics. Comprehensive analysis and discussion about target reflection and the BRDF model are included in previous works [33,35].

3D range gated reconstruction can be treated as one pixel problem where each pixel exhibits the same characteristics corresponding to the reflected laser pulse [36]. Thus, the same basic principles of the LADAR range equation apply. Target reflectivity ρ and angular dispersion $\theta_{R}$ correspond to the target reflection characteristics. Considering the effect of target reflection, we substitute the BRDF model Equation (12) into the LADAR Equation (10).

(13) $$P_{r}=\frac{\pi \eta_{sys}\eta_{atm}D^{2}}{4r^{2}}\left[\frac{K_{S}}{cos^{6}\theta }exp\left(\frac{-tan^{2}\theta }{s^{2}}\right)+K_{D}cos^{m}\theta \right]P_{t}.$$

The temporal function of the transmitted laser pulse $P_{t}\left(t\right)$ is commonly assumed as Gaussian [12]:

(14) $$P_{t}\left(t\right)=\frac{P_{o}}{\sqrt{2\pi }\sigma_{p}}exp\left(\frac{-t^{2}}{2\sigma_{p}^{2}}\right),$$

where $P_{o}$ is the transmitted power and $\sigma_{p}$ is the standard deviation of the echo pulse. From Equations (13) and (14), the reflected laser energy received $P_{r}\left(t\right)$ is written as:

(15) $$P_{r}\left(t\right)=\frac{\pi \eta_{sys}\eta_{atm}D^{2}}{4r^{2}}\left[\frac{K_{S}}{cos^{6}\theta }exp\left(\frac{-tan^{2}\theta }{s^{2}}\right)+K_{D}cos^{m}\theta \right]\frac{P_{o}}{\sqrt{2\pi }\sigma_{p}}exp\left(\frac{-t^{2}}{2\sigma_{p}^{2}}\right).$$

Based on the time slicing technique used for 3D reconstruction, the summation of radiant energy in an image pixel $I\left(x,y\right)=\sum_{i}I_{i}\left(x,y\right)$ can be equal to the integration over the time slices $\int dt_{i}/t_{step}$ as the time step $t_{step}$ is much smaller than width of laser pulse and camera gate [12]:

(16) $$I\left(x,y\right)=\sum_{i}I_{i}\left(x,y\right)=\frac{\int I_{i}\left(x,y\right)dt_{i}}{t_{step}}.$$

From Equation (7), we further obtain I(x,y) as:

(17) $$I\left(x,y\right)=\frac{\int P_{r}\left(x,y,t\right)dt\int G\left(\tau \right)d\tau }{t_{step}}.$$

By substituting $P_{r}\left(x,y,t\right)$ from Equation (15) and assuming G(τ)=1 when $0\leq \tau \leq t_{gate}$, I(x,y) is expressed as:

(18) $$I\left(x,y\right)=\frac{\pi \eta_{sys}\eta_{atm}D^{2}}{4r^{2}t_{step}}\left[\frac{K_{S}}{cos^{6}\theta }exp\left(\frac{-tan^{2}\theta }{s^{2}}\right)+K_{D}cos^{m}\theta \right]\frac{P_{o}}{\sqrt{2\pi }\sigma_{p}}\int_{-\infty }^{\infty }exp\left(\frac{-t^{2}}{2\sigma_{p}^{2}}\right)dt\int_{0}^{t_{gate}}d\tau .$$

We further simplify I(x,y) into:

(19) $$I\left(x,y\right)=\frac{\pi \eta_{sys}\eta_{atm}D^{2}}{4r^{2}}\left[\frac{K_{S}}{cos^{6}\theta }exp\left(\frac{-tan^{2}\theta }{s^{2}}\right)+K_{D}cos^{m}\theta \right]P_{o}\frac{t_{gate}}{t_{step}}.$$

From Equation (19), the camera gate $t_{gate}$ and time step $t_{step}$ are fixed during the images acquisition. The compensation factor α to the intensity received in an image pixel can be written as:

(20) $$\alpha ={\left(\frac{\pi \eta_{sys}\eta_{atm}D^{2}}{4r^{2}}\left[\frac{K_{S}}{cos^{6}\theta }exp\left(\frac{-tan^{2}\theta }{s^{2}}\right)+K_{D}cos^{m}\theta \right]\right)}^{-1}.$$

Average two-way travel time for an image pixel <t>(x,y) based on the intensity captured over times slices can be obtained by considering the compensation factor α:

(21) $$<t>\left(x,y\right)=\frac{\sum_{i=1}^{n}{\left(\frac{\pi \eta_{sys}\eta_{atm}D^{2}}{4r^{2}}\left[\frac{K_{S}}{cos^{6}\theta }exp\left(\frac{-tan^{2}\theta }{s^{2}}\right)+K_{D}cos^{m}\theta \right]\right)}^{-1}I_{i}\left(x,y\right)t_{i}}{\sum_{i=1}^{n}{\left(\frac{\pi \eta_{sys}\eta_{atm}D^{2}}{4r^{2}}\left[\frac{K_{S}}{cos^{6}\theta }exp\left(\frac{-tan^{2}\theta }{s^{2}}\right)+K_{D}cos^{m}\theta \right]\right)}^{-1}I_{i}\left(x,y\right)}.$$

Under the same set-up condition, system efficiency $\eta_{sys}$, atmospheric transmission loss factor $\eta_{atm}$ caused by absorption and scattering, and receiver parameter *D* can be regarded as constants, which can be ignored to further simplify the equation. On the other hand, range-squared factor $r^{2}$ is a variable across the time slices. Target reflection could vary depending on the characteristics of the target scene adhering to the BRDF model. Therefore, Equation (21) can be written as:

(22) $$<t>\left(x,y\right)=\frac{\sum_{i=1}^{n}{\left[\frac{K_{S}}{cos^{6}\theta }exp\left(\frac{-tan^{2}\theta }{s^{2}}\right)+K_{D}cos^{m}\theta \right]}^{-1}r_{i}^{2}I_{i}\left(x,y\right)t_{i}}{\sum_{i=1}^{n}{\left[\frac{K_{S}}{cos^{6}\theta }exp\left(\frac{-tan^{2}\theta }{s^{2}}\right)+K_{D}cos^{m}\theta \right]}^{-1}r_{i}^{2}I_{i}\left(x,y\right)}.$$

Equation (22) can be further simplified where variable $r_{i}$ corresponds to the range value for the particular *i*th time slice at delayed time $t_{i}$:

(23) $$<t>\left(x,y\right)=\frac{\sum_{i=1}^{n}{\left[\frac{K_{S}}{cos^{6}\theta }exp\left(\frac{-tan^{2}\theta }{s^{2}}\right)+K_{D}cos^{m}\theta \right]}^{-1}{\left(\frac{ct_{i}}{2}\right)}^{2}I_{i}\left(x,y\right)t_{i}}{\sum_{i=1}^{n}{\left[\frac{K_{S}}{cos^{6}\theta }exp\left(\frac{-tan^{2}\theta }{s^{2}}\right)+K_{D}cos^{m}\theta \right]}^{-1}{\left(\frac{ct_{i}}{2}\right)}^{2}I_{i}\left(x,y\right)}.$$

Accordingly, we obtain average time <t>(x,y) and range <r>(x,y) as:

(24) $$<t>\left(x,y\right)=\frac{\sum_{i=1}^{n}{\left[\frac{K_{S}}{cos^{6}\theta }exp\left(\frac{-tan^{2}\theta }{s^{2}}\right)+K_{D}cos^{m}\theta \right]}^{-1}I_{i}\left(x,y\right)t_{i}^{3}}{\sum_{i=1}^{n}{\left[\frac{K_{S}}{cos^{6}\theta }exp\left(\frac{-tan^{2}\theta }{s^{2}}\right)+K_{D}cos^{m}\theta \right]}^{-1}I_{i}\left(x,y\right)t_{i}^{2}}.$$

(25) $$<r>\left(x,y\right)=\frac{c<t>(x,y)}{2}=\frac{\sum_{i=1}^{n}{\left[\frac{K_{S}}{cos^{6}\theta }exp\left(\frac{-tan^{2}\theta }{s^{2}}\right)+K_{D}cos^{m}\theta \right]}^{-1}I_{i}\left(x,y\right)r_{i}^{3}}{\sum_{i=1}^{n}{\left[\frac{K_{S}}{cos^{6}\theta }exp\left(\frac{-tan^{2}\theta }{s^{2}}\right)+K_{D}cos^{m}\theta \right]}^{-1}I_{i}\left(x,y\right)r_{i}^{2}}.$$

For the distortion due to the inhomogeneous illumination, two assumptions are made. Firstly, the distortion ratio is unity at the centre of illumination, i.e., $r^{\prime }=r$ and the distortion effect increases as the image pixels (x,y) move radially from the centre. This radial distortion can be expressed as [37]:

(26) $$r^{d}=e+\lambda \left(r^{u}-e\right),$$

where $r^{d}$ and $r^{u}$ are the distorted and undistorted points, *e* indicates the centre of distortion, and λ denotes the distortion ratio. In our case, it is assumed that the centre of distortion equals to the centre of illumination, which is the image centre, i.e., e=0. *r* is considered as the distorted point $r^{d}$ and $r^{\prime }$ is the undistorted points $r^{u}$. Distortion ratio is equal to unity near the image centre to give $r=r^{\prime }$. This distortion between radial distance $r^{\prime }$ and orthogonal distance *r* can be modelled as:

(27) $$r=\lambda (r^{\prime }).$$

We propose the distortion ratio λ as a function of the angular difference between $r^{\prime }$ and *r*, which is bounded by the maximum diverging angle ϕ. λ can be modelled to decrease with respect to the angular difference θ(x,y) where 0<λ≤1.

(28) $$\lambda =\frac{r}{r^{\prime }}=cos\theta \left(x,y\right).$$

The distortion effect due to the inhomogeneous illumination can be regarded as radially symmetric because the lenses are typically ground to be circularly symmetric. Thus we make the second assumption that the distortion is radially symmetric within the illuminated area $\left(x_{max},y_{max}\right)$. θ(x,y) can be determined from the position of the image pixel (x,y) relative to the centre of illumination (0,0) and the maximum diverging angle ϕ:

(29) $$\theta \left(x,y\right)=\left(\frac{\sqrt{x^{2}+y^{2}}}{\sqrt{x_{max}^{2}+y_{max}^{2}}}\right)\phi .$$

Accordingly, we formulate the distortion ratio λ as:

(30) $$\lambda =cos\theta \left(x,y\right)=cos[\left(\frac{\sqrt{x^{2}+y^{2}}}{\sqrt{x_{max}^{2}+y_{max}^{2}}}\right)\phi ].$$

By considering the distortion correction, radial distance $r^{\prime }$ is obtained as follows from Equation (27):

(31) $$r^{\prime }=\frac{r}{\lambda }.$$

From Equations (25) and (30), the average range is then corrected as $<r^{\prime }>\left(x,y\right)$. Eventually, we propose a range estimation model as follows:

(32) $$<r^{\prime }>\left(x,y\right)=\frac{1}{\cos[\left(\frac{\sqrt{x^{2}+y^{2}}}{\sqrt{x_{max}^{2}+y_{max}^{2}}}\right)\phi ]}\frac{\sum_{i=1}^{n}{\left[\frac{K_{S}}{cos^{6}\theta }exp\left(\frac{-tan^{2}\theta }{s^{2}}\right)+K_{D}cos^{m}\theta \right]}^{-1}I_{i}\left(x,y\right)r_{i}^{3}}{\sum_{i=1}^{n}{\left[\frac{K_{S}}{cos^{6}\theta }exp\left(\frac{-tan^{2}\theta }{s^{2}}\right)+K_{D}cos^{m}\theta \right]}^{-1}I_{i}\left(x,y\right)r_{i}^{2}}.$$

## 5. Model Validation and Results Analysis

**Figure 5:**
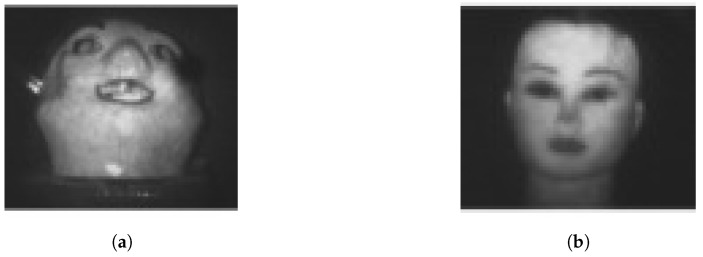
Raw image of the test objects captured by the range gated imaging system. (**a**) Raw image of Object 1. (**b**) Raw image of Object 2.

Based on the range gated imaging system specification, we can estimate the range accuracy using Equation (6) as explained in Section 3. Table 1 shows the experimental set-up specification used and the resulted range accuracy δr≈ 8.894 mm in the ideal scenario. The corresponding depth error can be calculated as:

(33) $$\%\mathrm{depth}\;\mathrm{error}=\frac{\delta r}{\mathrm{actual}\;\mathrm{object}\;\mathrm{depth}}\times 100\%.$$

where δr is the range accuracy or deviation of the reconstructed depth from the actual object depth reference which is measured manually with ruler. In our experimental study, Object 1 and Object 2 are tested where Object 1 has higher reflectivity relative to Object 2. Figure 5 shows the raw grayscale image of the test objects acquired using the range gated imaging system. The actual object depth of the test objects are 0.48 m and 0.4 m measured from background respectively. With the system range accuracy δr≈ 8.894 mm, it is estimated to give depth error ≈1.85% and 2.22% for Object 1 and Object 2, calculated from Equation (33). This unavoidable error calculated from the ideal experimental set-up specification is a rough estimation and deviation of the depth error is expected.

The range value for each pixel is calculated from the sequence of 2D images of the target scene using the conventional weighted average [12], range compensation [28], and the proposed range estimation model. From Equation (2), weighted average range is determined as:

(34) $$<r>\left(x,y\right)=\frac{c<t>(x,y)}{2}=\frac{\sum_{i=1}^{n}I_{i}\left(x,y\right)r_{i}}{\sum_{i=1}^{n}I_{i}\left(x,y\right)}.$$

Meanwhile, the range compensation model is expressed as:

(35) $$<r>\left(x,y\right)=\frac{\sum_{i=1}^{n}I_{i}\left(x,y\right)r_{i}^{3}}{\sum_{i=1}^{n}I_{i}\left(x,y\right)r_{i}^{2}}.$$

The proposed range estimation is calculated based on Equation (32). The calculated range for all image pixels eventually reconstruct the 3D surface of the test object. The 3D depth map shows the distance of the target scene to the camera. Accordingly, the reconstructed object depth can be determined from the difference between the maximum and minimum depth value. Absolute depth error between the reconstructed object depth and the actual object depth is calculated as follows:

(36) $$\%\mathrm{absolute}\;\mathrm{depth}\;\mathrm{error}=\frac{\left|\mathrm{reconstructed}\;\mathrm{object}\;\mathrm{depth}-\mathrm{actual}\;\mathrm{object}\;\mathrm{depth}\right|}{\mathrm{actual}\;\mathrm{object}\;\mathrm{depth}}\times 100\%.$$

**Figure 6:**
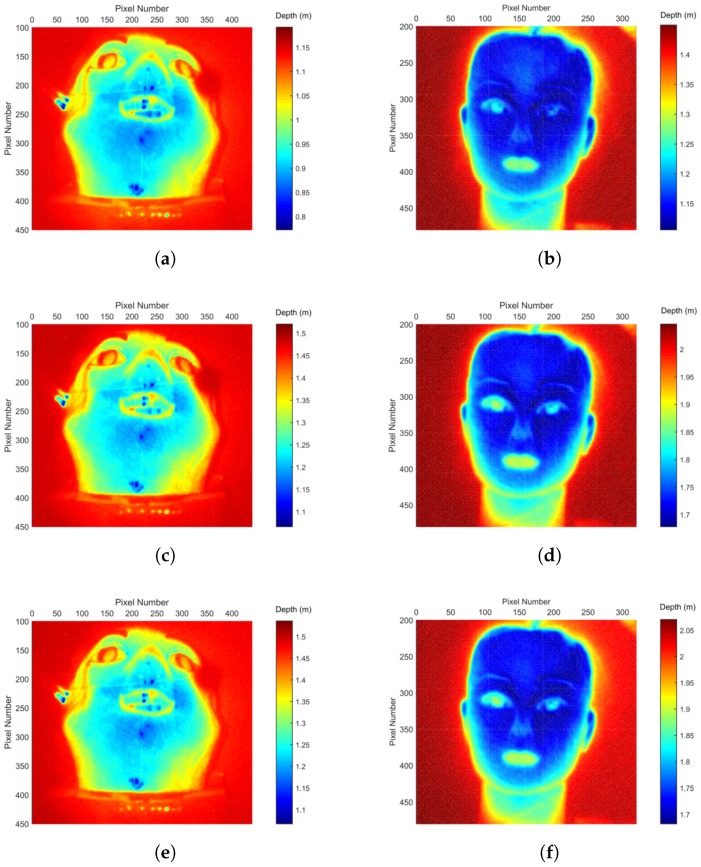
3D surface reconstruction based on the conventional weighted average, range compensation, and the proposed range estimation model for test objects. (**a**) Weighted average model for Object 1. (**b**) Weighted average model for Object 2. (**c**) Range compensation model for Object 1. (**d**) Range compensation model for Object 2. (**e**) Proposed range estimation model for Object 1. (**f**) Proposed range estimation model for Object 2.

The evaluation results are summarised in Table 2. Additionally, the results are compared to the estimated error per range gated imaging set-up specification in the ideal scenario. As the test objects have homogeneous surface material, target reflection BRDF compensation is not considered because the reflectivity is assumed to be uniform throughout the object surfaces. Based on the conventional weighted average method, depth errors of 12.65% and 14.11% are observed for Object 1 and Object 2. With the range compensation model proposed in previous work, the depth error of the objects are reduced to 5.42% and 8.11%. The proposed range estimation model further improves the reconstruction where the depth error reduces to 2.26% and 2.93% for Object 1 and Object 2, respectively. The proposed range estimation model results in smaller depth error as compared to the weighted average model that is commonly used and further refines the range estimation to give better accuracy than the range compensation model. Figure 6 shows the graphical comparison of 3D surface reconstruction based on weighted average, range compensation, and the proposed range estimation model for Object 1 and Object 2, respectively. As can be seen from Object 2, which is less reflective, the proposed model succeeds in reconstructing the eyebrows and the right eye. It also gives a uniform reconstruction in the neck area as compared to the weighted average method. The reconstructed background of the overall scene is more uniform as well. From the results, the proposed range estimation model outperforms the conventional weighted average and range compensation model to give a better range estimation for 3D range gated reconstruction.

**Figure 7:**
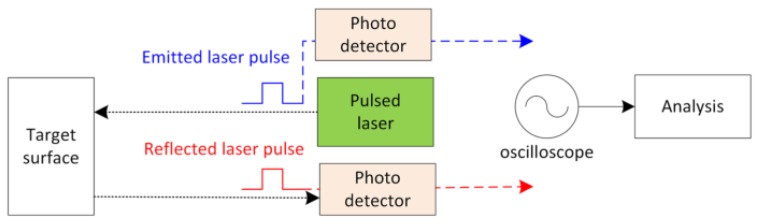
Schematic diagram of the experimental set-up for reflected laser investigation.

**Figure 8:**
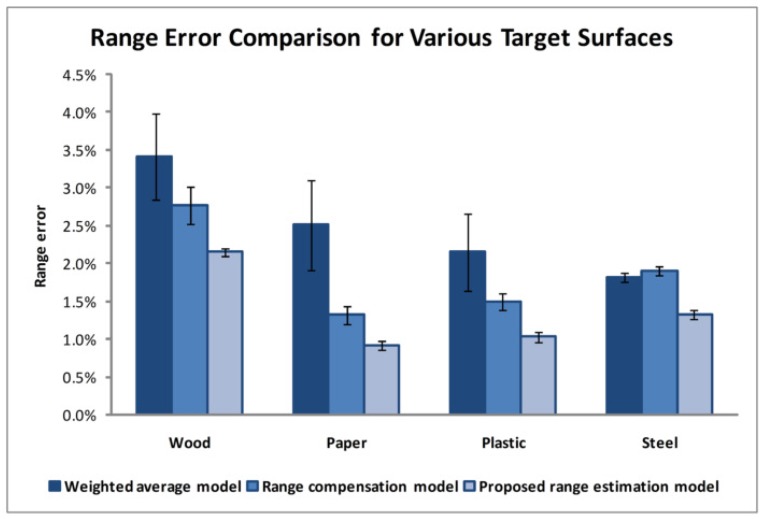
Range error comparison for various target surfaces calculated based on the conventional weighted average, range compensation, and the proposed range estimation model.

In addition, various target surface materials are tested. 3D range gated reconstruction can be treated as one pixel problem where each pixel exhibits the same characteristics corresponding to the reflected laser pulse [38]. Therefore, we perform an experimental study based on the reflected laser pulse using the set-up shown in Figure 7. The laser is emitted towards the flat target surface and returns a reflected signal. Photodetectors detect the emitted and reflected laser pulse to determine the range, which is the distance between the target surface and the photodetector. This experiment evaluates the ranging performance for different target surface materials based on the single pixel principle where some effects are excluded, for instance, the camera efficiency and noises. Results based on 30 measurements are summarised in Figure 8. The range error shown refers to the deviation between the calculated range and the actual range measured manually with ruler. The resulted range error using the proposed range estimation model is compared to the conventional weighted average [12] and range compensation model [28]. In general, the range error observed from the weighted average method is higher for a weak reflective target surface material such as wood. The range compensation model is able to improve the range determination but not for all cases. From the results, it can be seen that the proposed model consistently reduces the range error with lower relative standard error for the tested target surfaces.

## 6. Conclusions

Performance of 3D range gated reconstruction is strongly influenced by various factors in the system despite the advancement in lasers, sensors, signal processing, and computer technology. In this paper, detailed modelling of 3D range gated reconstruction and the range accuracy analysis are demonstrated to provide a comprehensive understanding of the influence factors and their impact to the system performance. Correspondingly, we propose a new range estimation model to address these influence factors to accomplish accurate reconstruction.

Based on the operating principle of time slicing technique, fundamental of radiant energy, LADAR, and BRDF, theoretical derivation of the range gated reconstruction model is presented. The derived model shows the relationship and dependency of various parameters with respect to the reflected laser intensity, SNR, and range accuracy. Accordingly, the algorithm of range estimation is improved by considering the energy attenuation and intensity variation due to distance and target reflection, and range distortion because of the inhomogeneous illumination. From the experimental results, the proposed range estimation model shows a noticeable improvement as compared to the conventional weighted average model, which proves the validity of the formulation presented. By comparing the results to the accuracy estimated from the set-up specification, the proposed model is able to achieve comparable performance.

In future, the proposed model can be a reference for ranging improvement, which contributes to miscellaneous applications. The range gated reconstruction presented in this study strongly relies on the reflectivity and range/time information associated with the images captured. Therefore, this is not suitable for non-reflective target and dark surface where weak or lack of laser intensity return will be encountered. For example, a black object absorbs the incoming laser and does not reflect any.

## Figures and Tables

**Table 1:** Range accuracy estimation based on the experimental letup specification.

Specification/Parameter	Value
Laser pulse width	4 ns
Camera gate time	5 ns
σ	≈9 ns
$t_{step}$	100 ps
$max(I_{i})$	$2^{8}$
SNR	≈151.79
δr	≈8.894 mm

**Table 2:** Absolute depth error (%) calculated based on the conventional weighted average, range compensation, and the proposed range estimation model as compared to the estimated depth error per set-up specification in ideal scenario.

Test Object	Depth Error per	Weighted Average	Range Compensation	Proposed Range
	Setup Specification	Model	Model	Estimation Model
Object 1	1.85%	12.65%	5.42%	2.26%
Object 2	2.22%	14.11%	8.11%	2.93%
